# Solid-State Point Defects through a Molecular Lens

**DOI:** 10.1021/acscentsci.6c01450

**Published:** 2026-09-08

**Authors:** Yu Jin, Timothy C. Berkelbach

**Affiliations:** † Initiative for Computational Catalysis, 525571 Flatiron Institute, New York, New York 10010, United States; ‡ Department of Chemistry, 5798 Columbia University, New York, New York 10027, United States

## Abstract

An embedded-cluster approach brings molecular quantum chemistry to solid-state diamond color centers and points to alternatives beyond the nitrogen-vacancy center.

## Graphical abstract

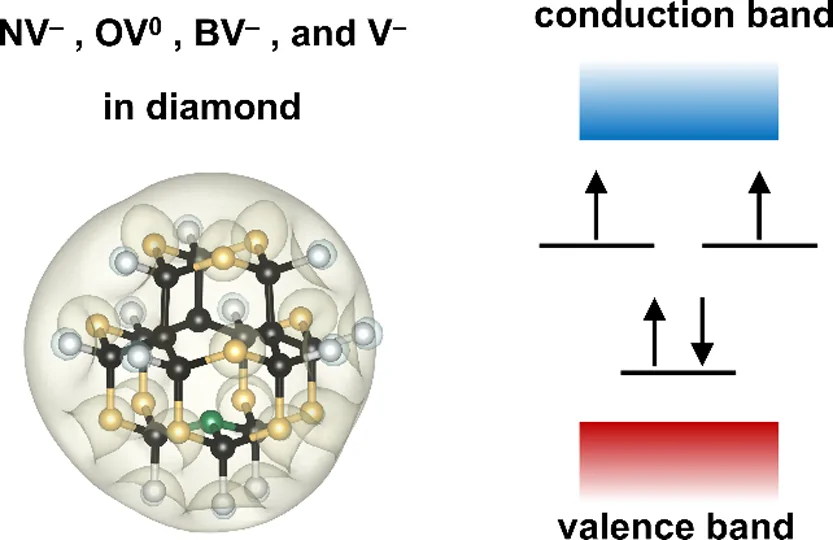

**O**ptically addressable point defects in solids, commonly known as color centers, have emerged as promising components for quantum sensing, communication, and computing.^1^ The negatively charged nitrogen-vacancy center in diamond is the most well-known, but many other point defects could be realized and potentially tailored for specific applications. The characterization and rational design of defects can be aided by accurate computer simulations, but the electronic structure of many defects is challenging for traditional methods, motivating many research efforts from the theoretical chemistry and physics communities.

Why are defect calculations challenging? On the one hand, the valence electrons can occupy several nearly degenerate, localized defect orbitals, demanding a multiconfigurational electronic structure description, such as complete active space methods, which are commonly used to study molecular transition-metal complexes. On the other hand, the host lattice contains an enormous number of atoms, and large supercells with periodic boundary conditions must be used to eliminate spurious finite-size errors. Unfortunately, complete active space methods—such as the complete active space self-consistent field (CASSCF) method plus perturbation theory—cannot be scaled to the large number of atoms in appropriately sized supercells, and they are rarely available with periodic boundary conditions.

In a recent issue of *ACS Central Science*,^2^ John Mark P. Martirez presents an embedded correlated-wavefunction (CW) framework that overcomes the above challenges, and the author applies it to a family of diamond color centers.

Carbon vacancies, neighboring substitutions (boron, nitrogen, or oxygen), and a handful of nearby carbon atoms are represented as “vacancy-centered molecules” embedded in a quantum-mechanically derived one-electron potential that approximates the effects of the surrounding diamond lattice.

Multiconfigurational CW methods, specifically CASSCF plus *n*-electron valence state perturbation theory (NEVPT2), are then applied to calculate ground-state and excited-state properties, including the relative energies of the various spin states. The calculations identify negatively charged boron-vacancy, B_C_V_C_^–^, and neutral oxygen-vacancy, O_C_V_C_^0^, complexes as possible alternatives to the negatively charged nitrogen-vacancy center, N_C_V_C_^–^.

The framework begins with periodic density-functional theory (DFT), which provides the defect geometries and electronic environment. A local cluster containing the dopant, the undercoordinated carbon atoms around the vacancy, and nearby atoms is extracted and chemically capped. The resulting “molecule” is placed in an optimized embedding potential designed to capture the influence of the remaining crystal: specifically, the potential is constructed so that a DFT calculation of the molecule with the embedding potential will reproduce a periodic DFT calculation. The approximate DFT calculation is then upgraded to an accurate calculation using CASSCF+NEVPT2 (Figure 1).^3^

**Figure 1:**
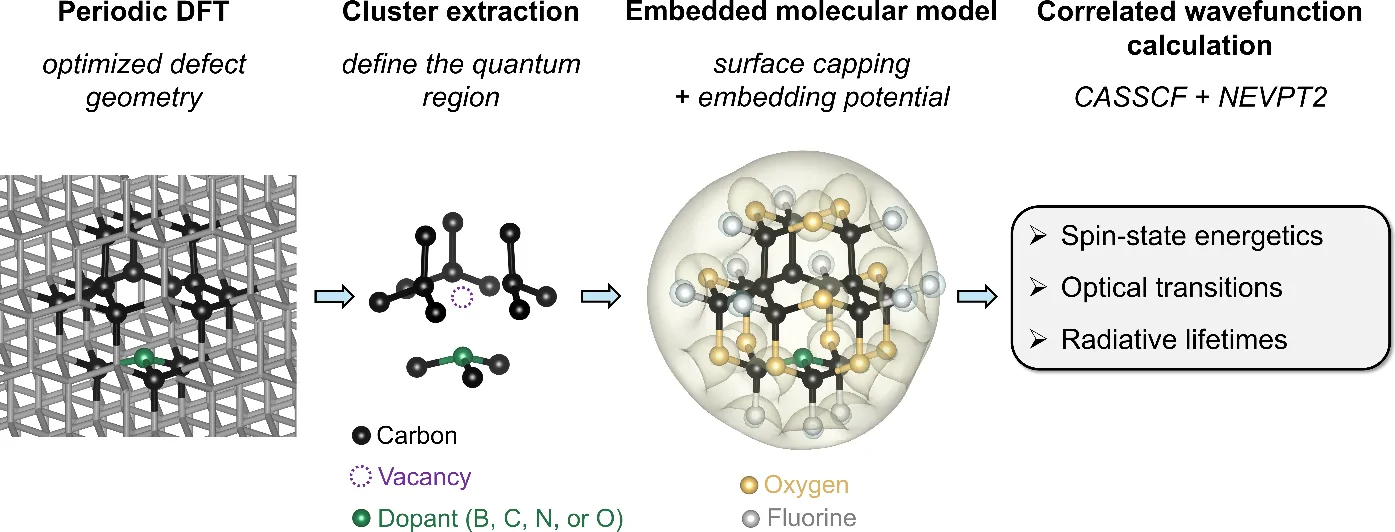
Embedded molecular quantum chemistry for diamond color centers. Periodic DFT provides the relaxed defect geometry and crystalline environment. A vacancy-centered region is extracted, chemically capped, and placed in an optimized embedding potential before multireference correlated-wavefunction calculations are performed.

This division of labor is well-suited to solid-state point defects. Periodic DFT supplies the optimized geometry and electronic influence of the crystalline environment, whereas the embedded CW treatment captures the multiconfigurational character of the localized defect states. Together, the two levels of theory enable calculations of spin-state energetics, optical transition energies and dipole moments, and radiative lifetimes that can be compared directly to experimental measurements.

Despite the sophistication of the computational method, the embedded molecular description provides a chemically intuitive basis for comparing the different defects, analogous to the description of open-shell transition metals using ligand-field theory, which is familiar to most chemists. The frontier orbitals are formed primarily from the undercoordinated carbon atoms surrounding the vacancy, while the neighboring dopant tunes their energies, occupations, and characters. Their symmetry accounts for the preferred spin states and Jahn-Teller distortions found for several charge states. This interpretation also suggests a chemical design principle: the vacancy provides the underlying orbital framework, while the dopant tunes its electronic structure.

The established N_C_V_C_^–^ center provides an important benchmark:^4^ its calculated spin state and optical properties agree well with available measurements, supporting the application of the same framework to less extensively characterized defects. The oxygen-vacancy complex is the closest electronic analog of N_C_V_C_^–^, but its bright transition is shifted from green to blue.^5^ The boron complex is more subtle: its brighter, N_C_V_C_^–^-like transition is not the lowest triplet excitation but the second, and it lies in the ultraviolet.^6^ Both candidates are predicted to have shorter radiative lifetimes than N_C_V_C_^–^ and nearby singlet states that may participate in shelving pathways.

The established N_C_V_C_^–^ center provides an important benchmark: its calculated spin state and optical properties agree well with available measurements, supporting the application of the same framework to less extensively characterized defects.

These results establish an accurate electronic structure foundation but connecting it to experimentally relevant optical cycles will require structural and dynamical information beyond vertical excitation energies. Zero-phonon lines, vibronic spectra, and nonradiative decay rates depend on how both the defect and the surrounding lattice reorganize after excitation. Their accurate calculation will require geometry optimization at the CW level for both ground and excited states, together with a consistent treatment of the response of the crystalline environment.^4,7^ Spin-orbit and electron-phonon couplings will be needed to translate accurate state energies into quantitative radiative and nonradiative rates, while the stability of the relevant atomic structures and charge states will determine whether the predicted optical cycles can be realized experimentally.

Although the embedded CW framework has made accurate calculations feasible, it is likely too expensive for routine use in high-throughput materials discovery. Rather, these calculations could serve as valuable benchmarks for more affordable methods or as a final, high-accuracy step in hierarchical screening workflows, after less expensive calculations have narrowed the candidate space. Its applicability beyond the present systems will depend on how well the embedded-cluster description performs when defect states are less localized and more strongly hybridized with the host bands, as may occur for some defects in smaller-gap semiconductors such as silicon.^8^ Whether the boron- and oxygen-vacancy centers ultimately become practical in quantum information applications remains for experiment to determine, but the embedded-molecule perspective offers a compelling way to identify candidates that merit further theoretical and experimental investigation.
